# *ALK*-rearrangements and testing methods in non-small cell lung cancer: a review

**DOI:** 10.18632/genesandcancer.3

**Published:** 2014-01

**Authors:** Rodney E. Shackelford, Moiz Vora, Kim Mayhall, James Cotelingam

**Affiliations:** ^1^ LSU Health Shreveport, Department of Pathology, Shreveport, LA, USA; ^2^ Tulane University School of Medicine, New Orleans, LA, USA

**Keywords:** Anaplastic lymphoma kinase, non-small cell lung cancer, pulmonary adenocarcinoma, crizotinib

## Abstract

The anaplastic lymphoma tyrosine kinase (ALK) gene was first described as a driver mutation in anaplastic non-Hodgkin's lymphoma. Dysregulated ALK expression is now an identified driver mutation in nearly twenty different human malignancies, including 4-9% of non-small cell lung cancers (NSCLC). The tyrosine kinase inhibitor crizotinib is more effective than standard chemotherapeutic agents in treating ALK positive NSCLC, making molecular diagnostic testing for dysregulated ALK expression a necessary step in identifying optimal treatment modalities. Here we review ALKmediated signal transduction pathways and compare the molecular protocols used to identify dysregulated ALK expression in NSCLC. We also discuss the use of crizotinib and second generation ALK tyrosine kinase inhibitors in the treatment of ALK positive NSCLC, and the known mechanisms of crizotinib resistance in NSCLC.

## INTRODUCTION

Lung cancer is the leading cause of cancer deaths world-wide [1]. Approximately 85% are non-small cell lung cancers (NSCLC), consisting mainly of squamous cell, adenocarcinoma, adenosquamous carcinoma, and large-cell anaplastic carcinoma, with most being adenocarcinomas [2,3]. Roughly 85% of lung cancers are caused by smoking, with the remaining related to factors such as individual genetics, and radon gas, asbestos, and air pollution exposures [2-4]. Most NSCLCs are diagnosed at an advanced stage, are clinically aggressive, and have a high metastatic potential. Thus NSCLC has a poor prognosis, with the majority of newly diagnosed individuals surviving less than one year and the five year survival rate being 16% [5]. Additionally, current NSCLC chemotherapeutic regimens have low efficacy. For example, patients with untreated advanced NSCLC have a median survival of 7.15 months, while those treated with current platinum-based doubled chemotherapy regimens have an 8-12 month median survival [4,5].

Over the past ten years intense research into the mechanisms of carcinogenesis and malignant progression has revealed that ~140 genes are altered in human malignancies, functioning as “driver mutations” that initiate and maintain malignancy [6]. While most adult malignancies carry 33-66 driver mutations, NSCLCs carry ~200 mutations, probably due to their arising in a background of cigarette smoke mutagen exposure [6]. Not surprisingly, lung cancers in non-smokers have 10-fold fewer mutations than those in smokers [7]. Presently, at least eighteen different driver mutations have been identified in NSCLC [8-38]. Thus, tumors once viewed as common “generic histologic types” are by molecular analysis composed of many tumor subtypes with similar histologies, but different molecular mechanisms of carcinogenesis and possible treatment modalities [38]. For example, epidermal growth factor receptor (EGFR]) gene mutations are found in 15-30% in NSCLCs and are an indication for tyrosine kinase inhibitor (TKI, erlotinib or gefitinib) therapy [38,39]. Several NSCLC mutations, such as the V600E and G479A BRAF mutations, are found in only 1-3% of NSCLCs and are tested for less frequently [38,40]. Most current molecular NSCLC testing is directed at EGFR, KRAS, and ALK mutation detection [38]. Specific therapeutic regimens exist for NSCLCs with EGFR, BRAF, and ALK mutations [38,39,41]. Presently KRAS mutations are undruggable, although benzimidazole compounds are being developed which inhibit oncogenic RAS signaling and suppress the *in vivo* and *in vitro* growth of pancreatic adenocarcinoma cells at nM concentrations [42].

Typically, the TKIs are used for locally advanced or metastatic NSCLC, or for NSCLC treatments that have failed standard chemotherapeutic regimens [43,44]. TKI therapy has little effect on malignancies lacking the driver mutation for specific TKI targets. For example, in patient populations with NSCLC unselected for EGFR mutations, the response rate to EGFR mutation-directed TKI therapy is ~9% [45,46]. In NSCLC patients with EGFR mutations the response rates to erlotinib or gefitinib are greater than 70% [47,48]. Based on this, molecular diagnostic testing for ALK and EGFR mutations is now recommended for NSCLCs to guide therapy [48,49]. Here we review the basic molecular pathology of ALK gene function in NSCLC, current testing methods, and review the current treatment strategies directed at ALK-mutation positive NSCLC.

### Anaplastic Lymphoma Kinase Gene Signaling

The anaplastic lymphoma kinase (ALK) gene is found at 2p23, spans 29 exons, and encodes a 1,620 amino acid, 220 kDa classical insulin superfamily tyrosine kinase. The mature ALK protein undergoes post-translational *N*-linked glycosylation and consists of an extracellular ligand-binding domain, a transmembrane domain, and a single intracellular tyrosine kinase domain. ALK is activated by dimerization with subsequent *trans*-autophosphorylation of three tyrosine moieties [50]. ALK is expressed in central and peripheral nervous systems, testes, skeletal muscle, basal layer keratinocytes, and small intestine. ALK appears to function in neuronal development and differentiation during embyrogenesis and its expression falls to low-levels at age three weeks and remains low throughout adult life [50-56]. Little is known about normal physiologic ALK function and ALK−/− mice show age-related increases in hippocampal progenitor cells, mild behavioral alterations, full viability, and have a normal lifespan [51,57]. In *D. melanogaster* and *C. elegans* the ALK-activating ligands Jelly belly and hesitation behavior have been identified, respectively [58,59]. In humans the heparin-binding growth factors Midkine and Pleiotrophin bind ALK have been reported to be the mammalian activating ligands [60,61]. However multiple studies have failed to confirm these results, so the endogenous ALK ligand remains controversial [20,62-65].

Activated ALK initiates several signal transduction pathways, including the Janus kinase, mammalian target of rapamycin, sonic hedgehog, phosphoinositide 3-kinase/protein kinase B, hypoxia-inducible factor-1α, JUNB, and phospholipase Cγ signaling. ALK signaling also regulates miR135b, mi29a, and miR-16, while Alk itself is regulated by miR-96 [50]. Analyses of ALK-signaling are complicated by the fact that different studies have employed different models, some of which examined wild-type ALK activity and others examining different ALK fusion protein activities. Thus, it's likely that some fusion protein targets do not represent “wild-type” or legitimate ALK phosphorylation targets [50].

### ALK Mutations in Cancer

ALK was first identified by Morris *et al*. (66) in anaplastic non-Hodgkin's lymphoma (ALCL), where it's fused to nucleophosmin, forming a t(2;5)(p23;q35) chromosomal translocation with constitutively active ALK kinase activity. Since this study activating ALK kinase mutations/translocations have been identified in a number of malignancies (Table 1) [8,9,50,56,66-97]. For many of these tumors, only a low percent are ALK positive [80,81]. ALK activation occurs largely through three different mechanisms: 1) fusion protein formation, 2) ALK over-expression, and 3) activating ALK point mutations [50]. While most histologically-defined tumor types have one of these mutation types, a few like the inflammatory myofibroblastic tumor (IMT) and NSCLC can have ALK mutations in two categories (Table 1). In the ALK translocations, the fusion partner regulates ALK expression levels, its subcellular location, and when it's expressed. Presently, there are 22 known different translocation partners that form fusion proteins with ALK [50]. In many cases, such as the echinoderm microtubule-associated protein-like 4 (EML4)-ALK fusion found in NSCLC, there are multiple fusion variants with different molecular weights, frequencies in NSCLC, protein stabilities (t1/2), and ALK inhibitor sensitivities [50,70,98,99] (Table 2). Rare individuals with non-functioning kinase ALK mutations have been identified. Presently it is unclear if these ALK “kinase dead” mutations promote tumor growth or are “passenger mutations” that do not effect cell proliferation [100].

**Table 1:** A list of the human malignancies known to express ALK dysregulated protein

Tumor Type	ALK Alteration	ALK Mutation Frequency	References
Anaplastic Non-Hodgkin's Lymphoma (ALCL)	t(2;5) ALK-Nucleophosmin chromosomal translocation	60-85% of ALCLs are ALK positive, rare fusions with TPM3, TPM4, TFG, ATIC, CLTC, MSN, MYH9, and ALO17	[66-69]
Non-Small Cell Lung Cancer	Chromosomal inversion/ translocation on 2p fusing EML4-ALK	3-7% of NSCLC are ALK positive, 21 known EML4-ALK breakpoints variants exist, rare fusions exist with TGF, KLC1, and KIKF5B	[8,9,50,70,71]
Basal Cell Carcinoma	ALK over-expression	250-fold increased phospho-ALK expression in ~100% of BCCs	[56]
Breast Cancer	EML4-ALK fusion	2.4% fusion positive by exon array profiling, 80% of inflammatory breast cancers show increased ALK protein	[72,73]
Neuroblastoma	ALK over-expression	ALK over-expressed in over 50% of tumors, ~12.4% of tumors carry activating ALK point mutations which are also common in familial neuroblastoma	[74-76]
Colorectal Carcinoma	EML4-ALK fusion	2.4% fusion positive by exon array profiling	[72]
Inflammatory Myofibroblastic Tumor (IMT)	Several ALK fusion proteins	~50% of IMTs are ALK positive, fusion partners include CLTC, TPM3, TPM4, CLTC, CARS, ATIC, RANBP2, and SEC31L1. Activating ALK point mutations are also occur.	[50, 77-79]
Diffuse large B-Cell Lymphoma	Several ALK fusion proteins	Rare, with ~50 cases described. The most common fusions partners are CLTC and NPM.	[80,81]
Glioblastoma	ALK over-expression	ALK is over-expressed, lowering ALK expression decreased glioblastoma tumor growth	[51,52]
Renal Carcinomas	Several translocations	ALK translocations with EML4, TPM3, and VCL fusion partners, the translocations appear to occur at a low frequency	[83-87]
Esophageal Squamous Cell Carcinoma	TPM4–ALK fusion	TPM4–ALK fusion oncoprotein type 2 found in ~20% of cases	[88]
Ewing's Sarcoma	ALK over-expression	Moderate to high ALK positivity was found in the majority of tumors	[89,90]
Ovarian Cancer	ALK over-expression	ALK over-expressed in 2-4% of ovarian cancers, one stromal sarcoma carried a FN1-ALK fusion protein	[91]
Anaplastic Thyroid Carcinoma	ALK activating point mutations	L1198F and G1201E amino acid changes result in constitutive ALK kinase activation	[92]
Melanoma	ALK over-expression	6.9% of acral melanomas were ALK positive, ALK breakpoints suggest that translocations are present	[93]
Rhabdomyosarcoma	ALK over-expression	45% alveolar rhabdomyosarcomas examined were ALK positive. High ALK mRNA expression is a negative prognostic marker	[94-96]
Retinoblastoma	Increased ALK mRNA	2/2 retinoblastoma cell lines over-expressed ALK kinase domain mRNA	[89]
Extramedullary plasmacytoma	ALK over-expression	1 case in 46 extramedullary plasmacytomas was ALK positive by immunohistochemistry and FISH analysis	[97]

**Table 2:** A list of the ALK translocation found in NSCLC and some of the more common EML4-ALK fusion protein characteristics (modified from references 50,70,98,99)

ALK Fusion Variant	EML-ALK Translocation Nomenclature	Fusion Protein Characteristics (cell growth inhibition studies were done in Ba/F3 cells expressing each EML4-ALK variant)	Frequency in NSCLC
E13;A20	E13; A20 (variant 1), E13;ins69 A20	Cytoplasmic location, longer protein t_1/2_, cell growth inhibition at moderate ALK inhibitor concentrations	33%
E6a/b;A20	E6a/b;A20 (variants 3a/b)	Cytoplasmic and nuclear locations, longer protein t_1/2_, cell growth inhibition requires lower or moderate ALK inhibitor concentrations (v3a and 3b, respectively)	29%
E20;A20	E20; A20 (variant 2), E20;ins18A20	Cytoplasmic location, shorter protein t_1/2_, cell growth inhibition at higher ALK inhibitor concentrations	9%
E14;A20	E14;ins11del49A20 (variant 4'), E14;del12A20 (variant 7)	No data	3%
E18;A20	E18; A20 (variant 5')	No data	2%
E15;A20	E15 del19;del20A20 (variant 4)	No data	2%
E2;A20	E2; A20 &E2;ins117A20 (variant 5a/b)	No data	2%
E2;A20	E17;ins68A20	No data	1%
E6;A19	Not defined	No data	One example
KLC1–ALK	-	No data	Rare
PTPN3–ALK	-	No data	Rare
STRN–ALK	-	No data	Rare

### ALK Activity in NSCLC

ALK was first identified in NSCLC by Soda *et al.* and Rikova *et al.* [8,9]. Rikova *et al.* [9] used global phosphotyrosine analysis to examine 41 NSCLC cell lines and 150 NSCLC tumors. Phospho-tyrosine peptides from these samples were purified and analyzed for specific phosphotyrosine kinase patterns. Patterns were detected for EGFR, c-Met, PDGFR-α, ROS, DDR1, and EML4-ALK and TGF-ALK fusion protein activities, with 4.4% of the NSCLCs being ALK fusion protein positive. Soda *et al.* [8] employed a retroviral cDNA expression library derived from a lung adenocarcinoma specimen which was infected into murine fibroblasts. One clone corresponded to the amino portion of EML4 and the carboxy portion of human ALK. Of 75 NSCLCs later examined 5 (6.7%) carried this open reading frame [8]. The EML4-ALK fusion results from an inversion in the short arm of chromosome two, fusing the N-terminal domain of EML4 to the intracellular kinase domain of ALK (3' gene region), resulting in a constitutively active ALK tryrosine kinase [8].

ALK translocation positive lung tumors are often adenocarcinomas with a solid or acinar histology, and focal signet-ring cell features, that often occur in younger patients who are never or former/light smokers [8,101-104]. Although ALK fusion proteins can coexist with other lung cancer driver mutations, these molecular double-hits are rare [8,102-015]. This observation is not surprising as small interfering RNA silencing of the EML4-Alk fusion in cell lines inhibits cell growth more than 50%, indicating that the EML4-ALK fusion, by itself, is sufficient as a malignancy driver mutation [72]. Due to the high world-wide lung cancer incidence (1.4 million deaths/year), ALK fusion positive lung cancers constitute the largest ALK positive patient population, comprising ~70,000 individuals [106]. Retrospective studies indicate that ALK fusion positivity was not a favorable prognostic factor in NSCLC prior to crizotinib based therapy [107]. Interestingly, Kim *et al*. [108] found ALK expression in 11.9% (8/67) of primary NSCLCs and 25.4% (17/67) of metastatic lesions, indicating that metastatic progression can be associated changes in ALK expression. Last, advanced stage ALK-positive lung cancers may have a higher propensity for pleural and pericardial disease than lung cancers lacking ALK, KRAS, or EGFR mutations [109].

### Molecular Diagnostic Testing Methods for ALK-Fusions in NSCLC

Since the introduction of crizotinib based chemotherapy, ALK mutation testing is now recommended for all NSCLCs [49]. There are several molecular ALK mutation testing methods; the most common are immunohistochemistry (IHC), fluorescent *in situ* hybridization (FISH), and polymerase chain reaction based techniques (PCR). Here we will briefly review these methods and their relative advantages and disadvantages.

#### FISH

FISH analysis is considered the Gold Standard for ALK NSCLC mutation testing. In 2011 FDA approved the Abbot Vysis ALK Break Apart FISH Probe Kit for molecular diagnostic testing [110,111]. For the Vysis ALK procedure unstained tissue hybridized overnight with the ALK probe and is evaluated by fluorescence microscopy [102,110,112,113]. An ALK translocation is present when the ALK probe shows separated red and green fluorophores, or has loss of the green signal, in 15% or more of the cells examined. The green fluorophore binds the region 5' to ALK, while red binds to the 3' ALK kinase encoding region [110,111]. FISH analysis for EML4-ALK translocations can be challenging as: 1) it has a high cost, 2) its accurate interpretation requires expertise and experience, 3) it does not identify specific translocation types, and 4) often has a lengthy turn-around time [102,110,112,113]. The advantages of FISH are that it should detect all ALK rearrangements regardless of the fusion partner and is accurate and reliable.

#### ALK IHC

IHC readily identifies ALK in ALCL [113,114]. However, ALK protein levels in ALK-rearranged NSCLCs are comparatively low, making the ALK IHC detection methods used for ALCL inadequate [115]. Additionally, there is currently no standard protocol for using IHC to detect ALK in NSCLC ([115,116]. Antibodies used with some success have been D5F3 (Cell Signaling Technology, Danvers, MA, USA), 5A4 (Novocastra, Newcastle, UK), and ALK clone ZAL4 (Invitrogen, Carlsbad, CA, USA) [73,83,86,88]. Thunnissen *et al*. [116] pointed out that the current challenges in developing IHC for ALK detection in NSCLC are: 1) tissue preparation, 2) antibody choice, 3) signal enhancement systems, and 4) the optimal scoring system. The main advantages of IHC are: 1) low cost, 2) relative ease of implementation, 3) ease of interpretation by the general pathologist, 4) retention of histologic information, and 5) a short turn-around time. Currently the clinical application ALK IHC in NSCLC requires further analysis and validation [102,112,115-117].

#### PCR

PCR-based techniques can detect ALK expression in NSCLC, with protocols including reverse-transcriptase multiplexed PCR and analyses of the relative expression of the 5' and 3' portions of the ALK gene transcript by RT-PCR [103,111,113,118,119].

### a. Reverse-Transcriptase PCR (RT-PRC)

RT-PCR is a precise, sensitive, and reproducible technique that can detect EML4-ALK fusion transcripts. Additionally, the amplicons can be sequenced to identify the specific fusion variants [102,112,118]. In this procedure RNA is converted into cDNA by reverse transcriptase and the cDNA is PCR amplified with specific primers. Amplification requires primer sets specific for each translocation [102,112,118]. Commercial kits are available which usually have primers to most or all of the EML4-ALK fusions transcripts [103,111,113,118,119]. The amplicons are identified by a variety of methods, including sequencing, fluorescent probe degradation, electrophoresis, and NanoString nCounter capture technology [102,110,112,118,120].

### b. 5'-Rapid Amplification of cDNA Ends (RACE) Analysis

All EML4-ALK fusion proteins carry the tyrosine kinase domain encoded by exon 20 and following distal 3' exons [69]. Wang *et al.* [91] used RACE analysis to quantify relative 5' and 3' ALK mRNA levels in NSCLCs. EML4-ALK mRNA was reverse transcribed into cDNA and different portions of the cDNA were amplified with primer sets specific to exons between E13-E18 and E22-E27. The E22-E27 domains where increased in 22.6% (40/177) of NSCLCs, ranging from 32.2 to 1573.7-fold increased expression when normalized to 5' ALK mRNA expression. PCR ALK analysis in NSCLC: 1) is specific and sensitive, 2) can detect the EML4-ALK fusion transcript diluted in over 90% wild-type RNA, and 3) is less expensive than FISH. The disadvantages of PCR are that: 1) it misses rare or novel translocations, 2) it can have contamination issues, and 3) RNA degradation/poor sample quality can prevent detection [102,110,112,118,120].

### Other Methods of EML4-ALK Detection in NSCLC

The EML4-ALK translocation has been detected by other less commonly employed molecular diagnostic methods, including:

### a. Next Generation Sequencing

Peled *et al.* [121] used comprehensive genomic profiling by second generation sequencing to identify a complex ALK rearrangement in a lung adenocarcinoma previously found to be EML4-ALK negative by the Vysis FISH assay. Sequencing revealed a complex ALK rearrangement involving at least five different genomic loci. Sequencing the cDNA derived from the complex rearrangement did reveal the canonical EML4-ALK breakpoint. The authors hypothesized that the EML4 and ALK genes were separated by small rearrangements that prevented detection by FISH assay. The tumor was also ALK positive by IHC and the patient responded to crizotinib therapy. The authors suggested that second generation sequencing may be useful for NSCLC patients with a high likelihood of harboring driver mutations not detected by other methods.

### b. Exon Array Profiling for EML4-ALK Fusions

Lin *et al.* [72] used exon array profiling (Affymetrix Human Exon 1.0 Arrays) to detect ALK rearrangements in breast, colorectal, and NSCLCs. Potential gene fusion candidates showed discordant 5' and 3' ALK transcript expression. Bioinformatic analysis revealed some tumors with differences between 5' and 3' ALK exon expression. Examination of these samples revealed EML4-ALK fusions in 2.4% of breast cancers (5/209), 2.4% of colorectal (2/83), and 11.3% of NSCLCs (12/106). Thus, while a complex, expensive, and technically challenging method, exon array profiling detects EML4-ALK fusions.

### Alk-Inhibitor-Specific Therapy for NSCLC

Following identification of the NSCLC EML4-Alk fusion, a search for effective inhibitors with clinical applications began. The first clinically useful inhibitor PF-2341066 (crizotinib), is now in widespread use for treating EML4-ALK fusion positive NSCLC [94,95]. Crizotinib is an orally active aminopyridine derived small-molecule ATP-competitive inhibitor with dual actions on the c-Met and ALK kinases. It was first identified as an ALK inhibitor in cell-based selectivity assays, where it exerts a half maximal inhibitory concentration at 24 nmol/L in NPM-ALK positive ALCL cell lines and showed a nearly 20-fold increased selectivity for the ALK and MET kinases compared to a panel of more than 120 different kinases [95]. Crizotinib induces a G1/S phase cell cycle checkpoint and apoptosis in ALK-rearrangement positive, but not negative lymphoma cells. SCID-Beige mice xenografted subcutaneously with NPM-ALK positive cells treated with crizotinib at 100mg/kg/day showed complete tumor regression within fifteen days, a significant tumor apoptosis induction, and a concomitant reduction in NPM-ALK phosphorylation and downstream signaling events [122-124].

The pharmacokinetics of crizotinib was first determined in humans in a Phase I clinical trial involving 167 patients who received an FDA-approved 250 mg dose BID [125]. Peak drug plasma concentrations were achieved in 4-6 hours and steady-state concentrations were reached in fifteen days. Crizotinib was widely distributed to most tissues, but exhibited poor the blood-brain barrier penetration. Its bioavailability was 43%, with 91% being protein bound. The side effects of crizotinib were documented in a different Phase I study began in May, 2006. Thirty-seven patients with advanced stage tumors including colorectal, pancreatic, sarcoma, ALCL, and NSCLCs, were enrolled in dose-escalation testing. Crizotinib was administered under fasting conditions QD or BID on a continuous schedule to the patients in successive dose-escalating cohorts, at doses ranging from 50 mg QD to 300 mg BID. Dose-limiting toxicities included grade 3 increased alanine aminotransferase and grade 3 fatigue. The most common mild (grade 1 or 2) side effects were nausea, emesis, fatigue and diarrhea, reversed with drug cessation [126].

One of the first large trials examining the effectiveness of crizotinib was a multicenter trial of 82 ALK-rearrangement positive advanced NSCLCs screened from 1,500 patients with NSCLC. Most of the patients had received prior treatment. They were treated with a 250mg BID crizotinib dose in 28-day cycles. The patients were assessed for therapy response and adverse drug effects. The overall response rate was 57% (47/82 patients) with 46 confirmed partial responses and one confirmed complete response. Twenty-seven (33%) patients had stable disease. The estimated progression-free survival was 72% and the majority of the side effects were grade 1 or 2. The authors concluded that crizotinib treatment resulted in tumor shrinkage in the majority of ALK-positive NSCLCs [127].

In a later study crizotinib treatment was compared to second line chemotherapy (docetaxel and pemetrexed) [128]. Three hundred and forty-seven patients with locally advanced or metastatic ALK-positive lung cancers who had received one prior treatment, were given 250 mg oral crizotinib BID or intravenous chemotherapy with either pemetrexed or docetaxel every three weeks. The median progression-free survival was 7.7 months in the crizotinib group and three months in the pemetrexed or docetaxel-based treatment group. The response rate was 65% in the crizotinib group and 19.5% in the second line chemotherapy group. The patients receiving crizotinib reported a greater quality of life improvement and reduction in lung cancer symptoms compared to the chemotherapy group. The authors concluded that crizotinib is superior to standard chemotherapy for the treatment of advanced NSCLC with ALK-rearrangements. Possibly, NSCLCs carrying different EML-ALK translocations may respond to ALK inhibitor therapy with different sensitivities, patient response rates, and tumor burden reduction characteristics. Presently these studies have not been performed.

Crizotinib was Federal Food and Drug Administration (FDA) approved on August 26, 2011 - the first FDA-approved NSCLC personalized therapy in which treatment is determined by clinically validated ALK testing [49,111,129]. Approval came five years after the initial clinical trails, with accelerated approval based on the surrogate endpoint of overall response rate. Post-marketing requirements include *in vitro* studies to evaluating its effects on the CYP2B and CYP2C enzymes and further clinical trials further evaluate its side-effects [130]. The European Medical Agency (EMA) approved crizotinib on 7/19/2012 following further analysis of randomized data [131]. FDA and EMA drug approval guidelines are similar in their relative efficacy of drug analysis, risk evaluation, and analysis of the drug once it's entered clinical use [132].

### Molecular Mechanisms of Crizotinib Resistance

Although ALK-positive tumors generally respond to critzotinib therapy, most patients relapse due to the development of resistance. In two small studies consisting of 12 and 18 ALK-positive, critzotinib-treated relapsed individuals, the average time of relapse was 8.9 and 10.5 months, with ranges from 3.5 to 21.1, and 4 to 34 months, respectively [99,133]. Resistance mechanisms are usually alterations in the EML-ALK fusion sequence, increased rearranged ALK gene copy number, or the activation of other driver mutations [99,134]. In some cases two or more resistance mechanisms are found in the same tumor [134]. Interestingly, in these studies only 36 and 28% of the critzotinib resistance was due to secondary rearranged ALK gene mutations not found in the tumor at the initial diagnosis [99,134, respectively]. Most resistance mechanisms involve the increased activity of other driver mutations [99,134]. Examples of some of the known mechanisms of critzotinib resistance are summarized in Table 3 (Table 3) [99-133-137]. Comparison of the initial diagnostic tumor biopsy to the resistant tumor often, but not always, revealed that the resistance mechanism was not present in the diagnostic biopsy [99,134]. Doebele *et al.* [99] identified two initially ALK-positive tumors which became negative following critzotinib therapy. Several studies demonstrated that critzotinib resistant cells had increased markers of cell growth and division compared to critzotinib sensitive cells, including increased Ki-67 and phosphorylated EGFR, ALK, ERK, and STAT3 [99,134,135]. Interestingly, increased autophagy may also play a role in critzotinib resistance [137].

**Table 3:** Different mechanisms of crizotinib resistance “pm” denotes a point mutation.

Mechanism of Crizotinib Resistance	Comment(s)	References
G1269A EML-ALKpm	Gly→Ala reduces crizotinib binding ATP-binding pocket by steric hindrance	[99]
L1196M EML-ALKpm	Gatekeeper residue mutation blocks crizotinib binding	[99,133,134]
C1156Y EML-ALKpm	Alters ALK crizotinib binding cavity, reducing crizotinib-protein interactions	[99,133,134]
S1206Y EML-ALKpm	Lowers crizotinib-protein affinity by eliminating two H-bonds between crizotinib and the ALK binding site	[133,134,135]
L1152R EML-ALKpm	Mutation resistant to crizotinib and the structurally unrelated compound TAE684	[136]
G1202R EML-ALKpm	A mutation-specific strong H-bond pulls crizotinib out of the position found in the non-crizotinib resistant EML-ALK fusion gene	[133,135]
1151Tins	Thr insertion is predicted to alter ATP binding to ALK	[133]
ALK Copy Number Gain	Two cases; one with and one without an ALK mutation, 4-5-fold increased expression	[99]
EGFR Alterations	L585R mutation in one case, other cases often show in EGFR and EGFR amplification	[99,133,136]
KRAS Mutations	G12C and G12V activating KRAS mutations	[99]
c-Kit	5-fold c-KIT amplification	[133]
Increased Autophagy	Increased autophagy involves Akt/mTOR signaling, autophagy inhibitors can restore crizotinib sensitivity in cell lines	[137]
Unknown	Crizotinib resistance by increased expression of unknown oncogenic drivers	[133]

### Second Generation ALK Inhibitors

Since resistance occurs in most critzotinib-treated patients, efforts have been made to develop ALK inhibitors which overcome this resistance. Phase I studies have been completed on several of these drugs and Phase 11 and III studies are ongoing [138-145]. One drug, LDK378 (Novartis), has received breakthrough FDA approval [146].

#### AP26113

AP26113 (Ariad) is an orally-active TKI that inhibits native and rearranged EML-ALK fusion proteins and the T790M (but not native) EGFR protein [138]. *In vitro* studies revealed that AP26113 inhibited the native and F1174C, L1196M, S1206R, E1210K, F1245C, and G1269S EML-ALK fusions at IC50's of 14-269 nM [145]. In the BaF3 xenograft model AP26113 induced tumor regression in cells expressing the native, G1269S, and L1196M fusion proteins at 25, 50, and 50 mg/kg, respectively [145]. In one study of 15 patients, 8 with NSCLC (4 ALK+ critzotinib-resistant and 4 with TKI-refectory EGFR mutations) were treated with AP26113. No serious adverse events were seen at 120 mg/day [138]. Partial responses were seen in 4 of 4 ALK-positive patients in the Phase II expansion study [138]. Further Phase II studies on other molecular cohorts, such as individuals with ALK-positive critzotinib-resistant NSCLCs and T790M EGFR-positive NSCLCs are being planned [144].

#### LDK378

LDK378 is an orally active ALK inhibitor which induced EML-ALK positive tumor regression in xenograft models and exhibited a minimally 70-fold greater ALK inhibition when compared to other kinases [141]. In a Phase I study of 59 patients, 50 of which had ALK-positive NSCLC, of which 37 of these had received critzotinib therapy and 26 of which had progressed on this therapy, 81% of this group (21/26) responded to > 400 mg/day LDK378 [139]. The maximum tolerated LDK378 dose was 750 mg/day, with the side effects being diarrhea, vomiting, nausea, dehydration, and ALT elevation [141]. Phase II trails are underway with this compound [146].

#### RO5424802

RO5424802 (Roche) is an orally active ALK-specific kinase inhibitor which binds the ALK binding domain, inhibiting ALK at nM concentrations [142,143]. RO5424802 treatment inhibited the growth of cells expressing the native, and L1196M and C1156Y EML-ALK mutants and inhibited ALK and STAT3 phosphorylation, and lowered the levels of the STAT3-regulated proteins BCL3 and NNMT [142,143]. In a Phase I and II dose escalation study 70 Japanese patients with ALk-positive NSCLCs were treated with RO5424802 [140]. In Phase I of this study 24 patients received 20-300 mg RO5424802 twice daily. No dose-limiting toxicities or grade 4 adverse events were seen at 300 mg twice daily. In Phase II of this study 46 patients were treated with this dose and 43 (93.5%) of patients achieved an objective response, including 2 complete responses (4.3%) and 41 (89.1%) partial responses [140]. Serious side effects occurred in 5 patients (11%) which included decreased neutrophils and increased blood creatine phosphokinase [140]. Interestingly, cell lines expressing the EML-ALK fusion exposed *in vitro* to RO5424802 develop resistance-conferring mutations, suggesting that RO5424802-resistant tumors may appear in treated individuals [147].

### HSP90 Inhibitors and EML-ALK Positive Lung Cancer

Heat shock proteins (HSP) function as part of normal cellular stress responses that protect cells from lethal damage and in cancer their increased expression contributes to increased tumor growth, metastasis, and a worse prognosis [148,149]. HSP90 has been extensively studied in cancer and is required for the correct folding and stability of multiple oncogenic proteins, including EML-ALK [150]. Inhibition of normal HSP90 function induces fusion protein misfolding and subsequent degradation by the proteasome system [150,151]. Several HSP90 inhibitors, AUY922, IPI 504, and Ganetespib (Norvartis, Infinity, and Synta Pharmaceuticals, respectively) have shown some efficacy suppressing the growth of EML-ALK fusion expressing cell lines and in treating ALK-positive NSCLC patients in Phase II trials [151-155].

## CONCLUSION

EML4-ALK fusions are found in a low percentage of NSCLCs [8,9]. However, since lung cancer is a common malignancy, an estimated 70,000 cases of ALK positive NSCLC occur world-wide each year, comprising the most common ALK-positive human malignancy [1-5,8,9,106]. Currently crizotinib is the “drug of choice” for the NSCLCs [49,11,129]. The clinical response rates with crizotinib and the newer ALK inhibitors are roughly 30% more effective than conventional chemotherapeutic treatments [122-128,138-145]. Thus, although ALK-rearrangement targeted treatments offer a better treatment regimen, advanced ALK-rearranged NSCLC still carries a poor prognosis. Several improvements that are likely to be implemented to improve targeted ALK-positive NSCLC include:

### 1.Implementation of low cost, reliable, and sensitive ALK detections methods

ALK detection by FISH is an expensive, slow, and cumbersome detection method [102,110,112,113]. ALK detection by IHC will likely replace FISH, as it is easier to implement, costs less, requires less expertise to perform, and has a rapid turn-around time [116,156]. It's also likely that second and third generation DNA sequencing will become more common in molecular diagnostics, as the cost of sequencing continues to fall [157]. Whole-exon or whole-genome sequencing could identify most or all changes NSCLC DNA. As the number of “actionable” (treatable) mutations increases, sequencing would be a more efficient and cost-effective molecular testing method than multiple tests analyzing a single gene alteration.

### 2.The development of new inhibitors in lung cancer treatment

New ALK inhibitors which overcome crizotinib resistance will soon enter clinical use. [138-146]. Additionally, KRAS mutations are found in 15-30% of lung cancers and are presently undruggable, although KRAS inhibitor are being developed [38,42]. New driver mutation specific inhibitors would significantly improve lung cancer treatment options.

### 3.The use of multiple inhibitors and/or multi-targeted inhibitors

Katayama *et al.* [133] found that one crizotinib resistant tumor which carried three different molecular resistance mechanisms. Molecular diagnostics defining the specific constellation of molecular alterations characterizing each NSCLC (especially for resistant tumors), could allow a specific directed cancer treatment with the least amount of damage to non-neoplastic tissue [158,159].

Taken together, these developments would significantly increase NSCLC patient survival while lowering treatment-associated morbidity.

## References

[1] Ferlay J, Shin HR, Bray F, Forman D, Mathers C, Parkin DM (2010). Estimates of worldwide burden of cancer in 2008: GLOBOCAN 2008. Int J Cancer.

[2] Ettinger DS, Akerley W, Bepler G (2010). NCCN Non-Small Cell Lung Cancer Panel Members. Non-small cell lung cancer. J Natl Compr Canc Netw.

[3] Hsiao SH, Chung CL, Lee CM (2013). Suitability of Computed Tomography-Guided Biopsy Specimens for Subtyping and Genotyping of Non-Small-Cell Lung Cancer. Clin Lung Cancer.

[4] Alberg AJ, Brock MV, Ford JG, Samet JM, Spivack SD (2013). Epidemiology of lung cancer: Diagnosis and management of lung cancer, 3rd ed: American College of Chest Physicians evidence-based clinical practice guidelines. Chest.

[5] SEER Cancer Statistics Review (1973-2008).

[6] Vogelstein B, Papadopoulos N, Velculescu VE, Zhou S, Diaz LA, Kinzler KW (2013). Cancer genome landscapes. Science.

[7] Govindan R, Ding L, Griffith M, Subramanian J (2012). Genomic landscape of non-small cell lung cancer in smokers and never-smokers. Cell.

[8] Soda M, Choi YL, Enomoto M, Takada S (2007). Identification of the transforming EML4-ALK fusion gene in non-small-cell lung cancer. Nature.

[9] Rikova K, Guo A, Zeng Q (2007). Global survey of phosphotyrosine signaling identifies oncogenic kinases in lung cancer. Cell.

[10] Samuels Y, Wang Z, Bardelli A (2004). High frequency of mutations of the PIK3CA gene in human cancers. Science.

[11] Heinmoller P, Gross C, Beyser K (2003). HER2 status in non-small cell lung cancer: results from patient screening for enrollment to a phase II study of herceptin. Clin Cancer Res.

[12] Stephens P, Hunter C, Bignell G (2004). Lung cancer: intragenic ERBB2 kinase mutations in tumours. Nature.

[13] Ding L, Getz G, Wheeler DA (2008). Somatic mutations affect key pathways in lung adenocarcinoma. Nature.

[14] Malanga D, Scrima M, De Marco C (2008). Activating E17K mutation in the gene encoding the protein kinase AKT1 in a subset of squamous cell carcinoma of the lung. Cell Cycle.

[15] Davies H, Bignell GR, Cox C (2002). Mutations of the BRAF gene in human cancer. Nature.

[16] Sasaki H, Kawano O, Endo K (2006). Uncommon V599E BRAF mutations in Japanese patients with lung cancer. J Surg Res.

[17] Naoki K, Chen TH, Richards WG, Sugarbaker DJ, Meyerson M (2002). Missense mutations of the BRAF gene in human lung adenocarcinoma. Cancer Res.

[18] Marks JL, Gong Y, Chitale D (2008). Novel MEK1 mutation identified by mutational analysis of epidermal growth factor receptor signaling pathway genes in lung adenocarcinoma. Cancer Res.

[19] Onozato R, Kosaka T, Kuwano H, Sekido Y, Yatabe Y, Mitsudomi T (2009). Activation of MET by gene amplifi cation or by splice mutations deleting the juxtamembrane domain in primary resected lung cancers. J Thorac Oncol.

[20] Cappuzzo F, Marchetti A, Skokan M (2009). Increased MET gene copy number negatively aff ects survival of surgically resected non-small-cell lung cancer patients. J Clin Oncol.

[21] Beau-Faller M, Ruppert AM, Voegeli AC (2008). MET gene copy number in non-small cell lung cancer: molecular analysis in a targeted tyrosine kinase inhibitor naive cohort. J Thorac Oncol.

[22] (2012). Cancer Genome Atlas Research Network. Comprehensive genomic characterization of squamous cell lung cancers. Nature.

[23] Forbes SA, Bindal N, Bamford S, Cole C, Kok CY, Beare D (2011). COSMIC: mining complete cancer genomes in the Catalogue of Somatic Mutations in Cancer. Nucleic Acids Res.

[24] Kohno T, Takahashi M, Manda R, Yokota J (1998). Inactivation of the PTEN/MMAC1/TEP1 gene in human lung cancers. Genes Chromosomes Cancer.

[25] Marsit CJ, Zheng S, Aldape K (2005). PTEN expression in non-small-cell lung cancer: evaluating its relation to tumor characteristics, allelic loss, and epigenetic alteration. Hum Pathol.

[26] Soria JC, Lee HY, Lee JI (2002). Lack of PTEN expression in non-small cell lung cancer could be related to promoter methylation. Clin Cancer Res.

[27] Jin G, Kim MJ, Jeon HS (2010). PTEN mutations and relationship to EGFR, ERBB2, KRAS, and TP53 mutations in non-small cell lung cancers. Lung Cancer.

[28] Bass AJ, Watanabe H, Mermel CH (2009). SOX2 is an amplified lineage-survival oncogene in lung and esophageal squamous cell carcinomas. Nat Genet.

[29] Dutt A, Ramos AH, Hammerman PS (2011). Inhibitor-sensitive FGFR1 amplification in human non-small cell lung cancer. PLoS ONE.

[30] Hammerman PS, Sos ML, Ramos AH (2011). Mutations in the DDR2 kinase gene identify a novel therapeutic target in squamous cell lung cancer. Cancer Discov.

[31] Matsumoto S, Iwakawa R, Takahashi K (2007). Prevalence and specificity of LKB1 genetic alterations in lung cancers. Oncogene.

[32] Hussenet T, Dali S, Exinger J (2010). SOX2 is an oncogene activated by recurrent 3q26.3 amplifications in human lung squamous cell carcinomas. PLoS ONE.

[33] Weiss J, Sos ML, Seidel D, Peifer M, Zander T, Heuckmann JM (2010). Frequent and focal FGFR1 amplification associates with therapeutically tractable FGFR1 dependency in squamous cell lung cancer. Sci Transl Med.

[34] Bass AJ, Watanabe H, Mermel CH (2009). SOX2 is an amplified lineage-survival oncogene in lung and esophageal squamous cell carcinomas. Nat Genet.

[35] Hammerman PS, Hayes DN, Wilkerson MD (2012). Comprehensive genomic characterization of squamous cell lung cancers. Nature.

[36] Rikova K, Guo A, Zeng Q (2007). Global survey of phosphotyrosine signaling identifies oncogenic kinases in lung cancer. Cell.

[37] Takeuchi K, Soda M, Togashi Y (2012). RET, ROS1 and ALK fusions in lung cancer. Nat Med.

[38] Planchard D (2013). Identification of driver mutations in lung cancer: first step in personalized cancer. Target Oncol.

[39] Köhler J, Schuler M (2013). Afatinib, Erlotinib and Gefitinib in the First-Line Therapy of EGFR Mutation-Positive Lung Adenocarcinoma: A Review. Onkologie.

[40] Ohashi K, Sequist LV, Arcila ME (2012). Lung cancers with acquired resistance to EGFR inhibitors occasionally harbor BRAF gene mutations but lack mutations in KRAS, NRAS, or MEK1. Proc Natl Acad Sci U S A.

[41] Burgeiro A, Mollinedo F, Oliveira PJ (2013). Ipilimumab and vemurafenib: two different routes for targeting melanoma. Curr Cancer Drug Targets.

[42] Zimmermann G, Papke B, Ismail S (2013). Small molecule inhibition of the KRAS-PDEδ interaction impairs oncogenic KRAS signalling. Nature.

[43] Maemondo M, Inoue A, Kobayashi K, Sugawara S, Oizumi S, Isobe H, Gemma A, Harada M, Yoshizawa H, Kinoshita I (2010). North-East Japan Study Group. Gefitinib or chemotherapy for non-small-cell lung cancer with mutated EGFR. N Engl J Med.

[44] Shaw AT, Kim DW, Nakagawa K, Seto T (2013). Crizotinib versus chemotherapy in advanced ALK-positive lung cancer. N Engl J Med.

[45] Shepherd FA, Rodrigues Pereira J, Ciuleanu T, Tan EH, Hirsh V, Thongprasert S, Campos D, Maoleekoonpiroj S, Smylie M (2005). National Cancer Institute of Canada Clinical Trials Group. Erlotinib in previously treated non-small-cell lung cancer. N Engl J Med.

[46] Jänne PA, Gurubhagavatula S, Yeap BY (2004). Outcomes of patients with advanced non-small cell lung cancer treated with gefitinib (ZD1839, “Iressa”) on an expanded access study. Lung Cancer.

[47] Jackman DM, Miller VA, Cioffredi LA (2009). Impact of epidermal growth factor receptor and KRAS mutations on clinical outcomes in previously untreated non-small cell lung cancer patients: Results of an online tumor registry of clinical trials. Clin Cancer Res.

[48] West H, Lilenbaum R, Harpole D (2009). Molecular analysis-based treatment strategies for the management of non-small cell lung cancer. J Thorac Oncol.

[49] Lindeman NI, Cagle PT, Beasley MB (2013). Molecular testing guideline for selection of lung cancer patients for EGFR and ALK tyrosine kinase inhibitors: guideline from the College of American Pathologists, International Association for the Study of Lung Cancer, and Association for Molecular Pathology. J Mol Diagn.

[50] Hallberg B, Palmer RH (2013). Mechanistic insight into ALK receptor tyrosine kinase in human cancer biology. Nat Rev Cancer.

[51] Bilsland J. G (2008). Behavioral and neurochemical alterations in mice deficient in anaplastic lymphoma kinase suggest therapeutic potential for psychiatric indications. Neuropsychopharmacology.

[52] Hurley SP, Clary D, Copie V, Lefcort F (2006). Anaplastic lymphoma kinase is dynamically expressed on subsets of motor neurons and in the peripheral nervous system. J. Comp. Neurol.

[53] Vernersson E (2006). Characterization of the expression of the ALK receptor tyrosine kinase in mice. Gene Expr. Patterns.

[54] Weiss J. B (2012). Anaplastic lymphoma kinase and leukocyte tyrosine kinase: functions and genetic interactions in learning, memory and adult neurogenesis. Pharmacol. Biochem. Behav.

[55] Iwahara T (1997). Molecular characterization of ALK, a receptor tyrosine kinase expressed specifically in the nervous system. Oncogene.

[56] Ning H, Mitsui H, Wang CQ, Suárez-Fariñas M (2013). Identification of anaplastic lymphoma kinase as a potential therapeutic target in Basal Cell Carcinoma. Oncotarget.

[57] Weiss JB, Xue C, Benice T, Xue L, Morris SW, Raber J (2012). Anaplastic lymphoma kinase and leukocyte tyrosine kinase: functions and genetic interactions in learning, memory and adult neurogenesis. Pharmacol Biochem Behav.

[58] Reiner DJ, Ailion M, Thomas JH, Meyer BJ (2008). C. elegans anaplastic lymphoma kinase ortholog SCD-2 controls dauer formation by modulating TGF-beta signaling. Curr Biol.

[59] Ishihara T, Iino Y, Mohri A (2002). HEN-1, a secretory protein with an LDL receptor motif, regulates sensory integration and learning in Caenorhabditis elegans. Cell.

[60] Stoica GE, Kuo A, Aigner I (2001). Identification of anaplastic lymphoma kinase as a receptor for the growth factor pleiotrophin. J. Biol. Chem.

[61] Stoica GE, Kuo A, Powers C (2002). Midkine binds to anaplastic lymphoma kinase (ALK) and acts as a growth factor for different cell types. J. Biol. Chem.

[62] Mathivet T1, Mazot P, Vigny M (2007). In contrast to agonist monoclonal antibodies, both C-terminal truncated form and full length form of Pleiotrophin failed to activate vertebrate ALK (anaplastic lymphoma kinase)?. Cell Signal.

[63] Moog-Lutz C1, Degoutin J, Gouzi JY (2005). Activation and inhibition of anaplastic lymphoma kinase receptor tyrosine kinase by monoclonal antibodies and absence of agonist activity of pleiotrophin. J Biol Chem.

[64] Mourali J1, Bénard A, Lourenço FC (2006). Anaplastic lymphoma kinase is a dependence receptor whose proapoptotic functions are activated by caspase cleavage. Mol Cell Biol.

[65] Motegi A1, Fujimoto J, Kotani M, Sakuraba H, Yamamoto T (2004). ALK receptor tyrosine kinase promotes cell growth and neurite outgrowth. J Cell Sci.

[66] Morris SW, Kirstein MN, Valentine MB, Dittmer KG, Shapiro DN, Saltman DL, Look AT (1994). Fusion of a kinase gene, ALK, to a nucleolar protein gene, NPM, in non-Hodgkin's lymphoma. Science.

[67] Delsol G, Ralfkiaer E, Stein H, Wright D, Jaffe ES, Jaffe ES, Harris NL, Stein H (2001). Anaplastic large cell lymphoma: primary systemic (T/null cell type). World Health Organization (WHO) Classification of Tumours: Pathology and Genetics of Tumours of Haematopoietic and Lymphoid Tissues.

[68] Stein H, Foss HD, Durkop H (2000). CD30(+) anaplastic large cell lymphoma: a review of its histopathologic, genetic, and clinical features. Blood.

[69] Pulford K, Lamant L, Espinos E (2004). The emerging normal and disease-related roles of anaplastic lymphoma kinase. Cell Mol Life Sci.

[70] Heuckmann JM, Balke-Want H, Malchers F (2012). Differential protein stability and ALK inhibitor sensitivity of EML4-ALK fusion variants. Clin Cancer Res.

[71] Takeuchi K, Choi YL, Togashi Y (2009). KIF5B-ALK, a novel fusion oncokinase identified by an immunohistochemistry-based diagnostic system for ALK-positive lung cancer. Clin Cancer Res.

[72] Lin E, Li L, Guan Y, Soriano R (2009). Exon array profiling detects EML4-ALK fusion in breast, colorectal, and non-small cell lung cancers. Mol Cancer Res.

[73] Robertson FM, Petricoin Iii EF, Van Laere SJ (2013). Presence of anaplastic lymphoma kinase in inflammatory breast cancer. Springerplus.

[74] Lamant L, Pulford K, Bischof D (2000). Expression of the ALK tyrosine kinase gene in neuroblastoma. Am J Pathol.

[75] Wang M, Zhou C, Sun Q, Cai R, Li Y, Wang D, Gong L (2013). ALK amplification and protein expression predict inferior prognosis in neuroblastomas. Exp Mol Pathol.

[76] Mossé YP, Laudenslager M, Longo L (2008). Identification of ALK as a major familial neuroblastoma predisposition gene. Nature.

[77] Cook JR, Dehner LP, Collins MH (2001). Anaplastic lymphoma kinase (ALK) expression in the inflammatory myofibroblastic tumor: a comparative immunohistochemical study. Am J Surg Pathol.

[78] Gleason BC, Hornick JL (2008). Inflammatory myofibroblastic tumours: where are we now?. J Clin Pathol.

[79] Coffin CM, Patel A, Perkins S, Elenitoba-Johnson KS, Perlman E, Griffin CA (2001). ALK1 and p80 expression and chromosomal rearrangements involving 2p23 in inflammatory myofibroblastic tumor. Mod Pathol.

[80] Gascoyne RD, Lamant L, Martin-Subero JI (2003). ALK-positive diffuse large B-cell lymphoma is associated with Clathrin-ALK rearrangements: report of 6 cases. Blood.

[81] Laurent C, Do C, Gascoyne RD, Lamant L (2009). Anaplastic lymphoma kinase-positive diffuse large B-cell lymphoma: a rare clinicopathologic entity with poor prognosis. J Clin Oncol.

[82] Powers C, Aigner A, Stoica GE, McDonnell K, Wellstein A (2002). Pleiotrophin signaling through anaplastic lymphoma kinase is rate-limiting for glioblastoma growth. J Biol Chem.

[83] Grzelinski M, Steinberg F, Martens T, Czubayko F, Lamszus K, Aigner A (2009). Enhanced antitumorigenic effects in glioblastoma ondouble targeting of pleiotrophin and its receptor ALK. Neoplasia.

[84] Debelenko LV, Raimondi SC, Daw N (2011). Renal cell carcinoma with novel VCL-ALK fusion: new representative of ALK-associated tumor spectrum. Mod Pathol.

[85] Mariño-Enríquez A, Ou WB, Weldon CB, Fletcher JA, Pérez-Atayde AR (2011). ALK rearrangement in sickle cell trait-associated renal medul¬lary carcinoma. Genes Chromosomes Cancer.

[86] Sukov WR, Hodge JC, Lohse CM (2012). ALK alterations in adult re¬nal cell carcinoma: frequency, clinicopathologic features and out¬come in a large series of consecutively treated patients. Mod Pathol.

[87] Sugawara E, Togashi Y, Kuroda N (2012). Identification of anaplastic lymphoma kinase fusions in renal cancer: large-scale immunohis¬tochemical screening by the intercalated antibody-enhanced poly¬mer method. Cancer.

[88] Du XL, Hu H, Lin DC, Xia SH (2007). Proteomic profiling of proteins dysregulted in Chinese esophageal squamous cell carcinoma. J Mol Med.

[89] Dirks WG, Fahnrich S, Lis Y (2002). Expression and functional analysis of the anaplastic lymphoma kinase (ALK) gene in tumor cell lines. Int J Cancer.

[90] Li XQ, Hisaoka M, Shi DR (2004). Expression of anaplastic lymphoma kinase in soft tissue tumors an immunohistochemical and molecular study of 249 cases. Hum Pathol.

[91] Ren H, Tan ZP, Zhu X (2012). Identification of anaplastic lymphoma kinase as a potential therapeutic target in ovarian cancer. Cancer Res.

[92] Murugan AK, Xing M (2011). Anaplastic thyroid cancers harbor novel oncogenic mutations of the ALK gene. Cancer Res.

[93] Niu HT, Zhou QM, Wang F (2013). Identification of anaplastic lymphoma kinase break points and oncogenic mutation profiles in acral/mucosal melanomas. Pigment Cell Melanoma Res.

[94] Pillay K, Govender D, Chetty R (2002). ALK protein expression in rhabdomyosarcomas. Histopathology.

[95] Corao DA, Biegel JA, Coffin CM (2009). ALK expression in rhabdomyosarcomas: correlation with histologic subtype and fusion status. Pediatr Dev Pathol.

[96] Bonvini P, Zin A, Alaggio R, Pawel B, Bisogno G, Rosolen A (2013). High ALK mRNA expression has a negative prognostic significance in rhabdomyosarcoma. Br J Cancer.

[97] Wang WY, Gu L, Liu WP, Li GD, Liu HJ, Ma ZG (2011). ALK-positive extramedullary plasmacytoma with expression of the CLTC-ALK fusion transcript. Pathol Res Pract.

[98] Sasaki T, Rodig SJ, Chirieac LR, Jänne PA (2010). The biology and treatment of EML4-ALK non-small cell lung cancer. Eur J Cancer.

[99] Doebele RC1, Pilling AB, Aisner DL (2012). Mechanisms of resistance to crizotinib in patients with ALK gene rearranged non-small cell lung cancer. Clin Cancer Res.

[100] Schönherr C, Ruuth K, Eriksson T (2011). The neuroblastoma ALK(I1250T) mutation is a kinase-dead RTK in vitro and in vivo. Transl Oncol.

[101] Rodig SJ, Mino-Kenudson M, Dacic S (2009). Unique clinicopathologic features characterize ALK-rearranged lung adenocarcinoma in the western population. Clin Cancer Res.

[102] Li Y, Pan Y, Wang R, Sun Y (2013). ALK-rearranged lung cancer in Chinese: a comprehensive assessment of clinicopathology, IHC, FISH and RT-PCR. PLoS One.

[103] Shaw AT, Yeap BY, Mino-Kenudson M (2009). Clinical features and outcome of patients with non-small-cell lung cancer who harbor EML4-ALK. J Clin Oncol.

[104] Inamura K, Takeuchi K, Togashi Y (2008). EML4-ALK fusion is linked to histological characteristics in a subset of lung cancers. J Thorac Oncol.

[105] Shaozhang Z, Xiaomei L, Aiping Z (2012). Detection of EML4-ALK fusion genes in non-small cell lung cancer patients with clinical features associated with EGFR mutations. Genes Chromosomes Cancer.

[106] Jemal A, Clegg LX, Ward E (2004). Annual report to the nation on the status of cancer, 1975–2001, with a special feature regarding survival. Cancer.

[107] Shaw AT (2001). Impact of crizotinib on survival in patients with advanced, ALK-positive NSCLC compared with historical controls. J. Clin. Oncol.

[108] Kim H, Xu X, Yoo SB (2013). Discordance between anaplastic lymphoma kinase status in primary non-small-cell lung cancers and their corresponding metastases. Histopathology.

[109] Doebele RC, Lu X, Sumey C (2012). Oncogene status predicts patterns of metastatic spread in treatment-naive nonsmall cell lung cancer. Cancer.

[110] Lira ME, Kim TM, Huang D (2013). Multiplexed gene expression and fusion transcript analysis to detect ALK fusions in lung cancer. J Mol Diagn.

[111] http://www.abbott.com/news-media/press-releases/2011-aug26.htm.

[112] Wu YC, Chang IC, Wang CL (2013). Comparison of IHC, FISH and RT-PCR methods for detection of ALK rearrangements in 312 non-small cell lung cancer patients in Taiwan. PLoS One.

[113] Lamant L, Meggetto F, al Saati T (1996). High incidence of the t(2;5)(p23;q35) translocation in anaplastic large cell lymphoma and its lack of detection in Hodgkin's disease. Comparison of cytogenetic analysis, reverse transcriptase-polymerase chain reaction, and P-80 immunostaining. Blood.

[114] Pulford K, Lamant L, Morris SW (1997). Detection of anaplastic lymphoma kinase (ALK) and nucleolar protein nucleophosmin (NPM)-ALK proteins in normal and neoplastic cells with the monoclonal antibody ALK1. Blood.

[115] Mino-Kenudson M, Chirieac LR, Law K (2010). A novel, highly sensitive antibody allows for the routine detection of ALK-rearranged lung adenocarcinomas by standard immunohistochemistry. Clin Cancer Res.

[116] Thunnissen E, Bubendorf L, Dietel M (2012). EML4-ALK testing in non-small cell carcinomas of the lung: a review with recommendations. Virchows Arch.

[117] Paik JH, Choe G, Kim H, Choe JY (2011). Screening of anaplastic lymphoma kinase rearrangement by immunohistochemistry in non-small cell lung cancer: correlation with fluorescence in situ hybridization. J Thorac Oncol.

[118] Zhang YG, Jin ML, Li L (2013). Evaluation of ALK rearrangement in Chinese non-small cell lung cancer using FISH, immunohistochemistry, and real-time quantitative RT- PCR on paraffin-embedded tissues. PLoS One.

[119] Wang R, Pan Y, Li C, Hu H (2012). The use of quantitative real-time reverse transcriptase PCR for 5' and 3' portions of ALK transcripts to detect ALK rearrangements in lung cancers. Clin Cancer Res.

[120] Geiss GK, Bumgarner RE, Birditt B (2008). Direct multiplexed measurement of gene expression with color-coded probe pairs. Nat Biotechnol.

[121] Peled N, Palmer G, Hirsch FR (2012). Next-generation sequencing identifies and immunohistochemistry confirms a novel crizotinib-sensitive ALK rearrangement in a patient with metastatic non-small-cell lung cancer. J Thorac Oncol.

[122] Christensen JG, Zou HY, Arango ME (2007). Cytoreductive antitumor activity of PF-2341066, a novel inhibitor of anaplastic lymphoma kinase and c-Met, in experimental models of anaplastic large-cell lymphoma. Mol Cancer Ther.

[123] Cui J (2011). Structure based drug design of crizotinib (PF-02341066), a potent and selective dual inhibitor of mesenchymal–epithelial transition factor (c-MET) kinase and anaplastic lymphoma kinase (ALK). Journal of medicinal chemistry.

[124] Zou HY (2007). An orally available small-molecule inhibitor of c-Met, PF-2341066, exhibits cytoreductive antitumor efficacy through antiproliferative and antiangiogenic mechanisms. Cancer Res.

[125] Li C, Alvey C, Bello A (2011). Pharmacokinetics (PK) of crizotinib (PF-02341066) in patients with advanced non-small cell lung cancer (NSCLC) and other solid tumors. J Clin Oncol.

[126] Kwak EL, Camidge DR, Clark J (2009). Clinical activity observed in a phase I dose escalation trial of an oral c-met and ALK inhibitor, PF-02341066. J Clin Oncol 27:15s.

[127] Kwak EL, Bang YJ, Camidge DR (2010). Anaplastic lymphoma kinase inhibition in non-small-cell lung cancer. N Engl J Med.

[128] Shaw AT, Kim DW, Nakagawa K (2013). Crizotinib versus chemotherapy in advanced ALK-positive lung cancer. N Engl J Med.

[129] http://www.accessdata.fda.gov/scripts/cder/drugsatfda/index.cfm?fuseaction=Search.DrugDetails.

[130] Malik SM, Maher VE, Bijwaard KE (2014). U.S. Food and Drug Administration Approval: Crizotinib for Treatment of Advanced or Metastatic Non-small Cell Lung Cancer that Is Anaplastic Lymphoma Kinase Positive. Clin Cancer Res.

[131] http://www.valuebasedcancer.com/article/crizotinib-superior-chemotherapy-extends-median-survival-first-head-head-trial.

[132] Lis Y, Roberts MH, Kamble S, J Guo J, Raisch DW (2012). Comparisons of Food and Drug Administration and European Medicines Agency risk management implementation for recent pharmaceutical approvals: report of the International Society for Pharmacoeconomics and outcomes research risk benefit management working group. Value Health.

[133] Katayama R1, Shaw AT, Khan TM (2012). Mechanisms of acquired crizotinib resistance in ALK-rearranged lung Cancers. Sci Transl Med.

[134] Choi YL, Soda M, Yamashita Y (2010). EML4-ALK mutations in lung cancer that confer resistance to ALK inhibitors. N Engl J Med.

[135] Sun HY1, Ji FQ (2012). A molecular dynamics investigation on the crizotinib resistance mechanism of C1156Y mutation in ALK. Biochem Biophys Res Commun.

[136] Sasaki T, Koivunen J, Ogino A (2011). A novel ALK secondary mutation and EGFR signaling cause resistance to ALK kinase inhibitors. Cancer Res.

[137] Ji C, Zhang L, Cheng Y, Patel R (2014). Induction of autophagy contributes to crizotinib resistance in ALK-positive lung cancer. Cancer Biol Ther.

[138] Gettinger S, Weiss GJ, Salgia R (2012). A first in human dose finding study of the ALK/EGFR inhibitor AP26113 in patients with advanced malignanices. Ann Oncol.

[139] Shaw AT, Camidge DR, Felip E (2012). Results of the first human phase I study of the ALK inhibitor LKD378 in advanced solid tumors. Ann Oncol.

[140] Seto T, Kiura K, Nishio M, Nakagawa K (2013). CH5424802 (RO5424802) for patients with ALK-rearranged advanced non-small-cell lung cancer (AF-001JP study): a single-arm, open-label, phase 1-2 study. Lancet Oncol.

[141] Marsilje TH1, Pei W, Chen B (2013). Synthesis, structure-activity relationships, and in vivo efficacy of the novel potent and selective anaplastic lymphoma kinase (ALK) inhibitor 5-chloro-N2-(2-isopropoxy-5-methyl-4-(piperidin-4-yl)phenyl)-N4-(2-(isopropylsulfonyl)phenyl)pyrimidine-2,4-diamine (LDK378) currently in phase 1 and phase 2 clinical trials. J Med Chem.

[142] Kinoshita K1, Asoh K, Furuichi N (2012). Design and synthesis of a highly selective, orally active and potent anaplastic lymphoma kinase inhibitor (CH5424802). Bioorg Med Chem.

[143] Sakamoto H, Tsukaguchi T, Hiroshima S (2011). CH5424802, a selective ALK inhibitor capable of blocking the resistant gatekeeper mutant. Cancer Cell.

[144] Lazzari C, Spitaleri G, Catania C (2014). Targeting ALK in patients with advanced Non Small Cell Lung Cancer: Biology, diagnostic and therapeutic options. Crit Rev Oncol Hematol.

[145] Zhang S, Wang F, Keats J (2010). AP26113, a potent ALK inhibitor, overcomes mutations in EML4-ALK that confer resistance to PF-02341066 (PF1066). Cancer Research.

[146] http://clinicaltrials.gov/show/NCT01685060.

[147] Zdzalik D, Dymek B, Grygielewicz P (2014). Activating mutations in ALK kinase domain confer resistance to structurally unrelated ALK inhibitors in NPM-ALK-positive anaplastic large-cell lymphoma. J Cancer Res Clin Oncol.

[148] Garcia-Carbonero R, Carnero A, Paz-Ares L (2013). Inhibition of HSP90 molecular chaperones: moving into the clinic. Lancet Oncol.

[149] Mahalingam D, Swords R, Carew JS (2009). Targeting HSP90 for cancer therapy. Br J Cancer.

[150] Neckers L, Workman P (2012). Hsp90 molecular chaperone inhibitors: are we there yet?. Clin Cancer Res.

[151] Normant E, Paez G, West KA (2011). The Hsp90 inhibitor IPI-504 rapidly lowers EML4-ALK levels and induces tumor regression in ALK-driven NSCLC models. Oncogene.

[152] Ying W, Du Z, Sun L (2012). Ganetespib, a unique triazolone-containing Hsp90 inhibitor, exhibits potent antitumor activity and a superior safety profile for cancer therapy. Mol Cancer Ther.

[153] Sequist LV, Gettinger S, Senzer NN (2010). Activity of IPI-504, a novel heat-shock protein 90 inhibitor, in patients with molecularly defined nonsmall-cell lung cancer. J Clin Oncol.

[154] Socinski MA, Goldman J, El-Hariry I (2013). A multicenter phase II study of ganetespib monotherapy in patients with genotypically defined advanced non-small-cell lung cancer. Clin Cancer Res.

[155] Felip E, Carcereny E, Barlesi F (2012). Phase II activity of the Hsp90 inhibitor AUY922 in patients with ALK rearranged (ALK+) or EGFR mutated advanced non small cell lung cancer (NSCLC). Ann Oncol.

[156] Ying J, Guo L, Qiu T, Shan L, Ling Y, Liu X, Lu N (2013). Diagnostic value of a novel fully automated immunochemistry assay for detection of ALK rearrangement in primary lung adenocarcinoma. Ann Oncol.

[157] Morey M1, Fernández-Marmiesse A, Castiñeiras D (2013). glimpse into past, present, and future DNA sequencing. Mol Genet Metab.

[158] Broekman F, Giovannetti E, Peters GJ (2011). Tyrosine kinase inhibitors: Multi-targeted or single-targeted?. World J Clin Oncol.

[159] Looyenga BD, Cherni I, MacKeigan JP, Weiss GJ (2011). Tailoring Tyrosine Kinase Inhibitors to Fit the Lung Cancer Genome. Transl Oncol.

